# Sorption of BTEX on a nanoporous composite of SBA-15 and a calcined hydrotalcite

**DOI:** 10.1186/s40580-018-0153-2

**Published:** 2018-08-02

**Authors:** Alvaro Sampieri, Gabriela Pérez-Osorio, Miguel Ángel Hernández-Espinosa, Irving Israel Ruiz-López, Mayra Ruiz-Reyes, Janette Arriola-Morales, Rocío Iliana Narváez-Fernández

**Affiliations:** 1 Facultad de Ingeniería Química, Benemérita Universidad Autónoma de Puebla, Ciudad Universitaria, 72570 Puebla, PUE Mexico; 2 Departamento de Investigación en Zeolitas del Instituto de Ciencias, Benemérita Universidad Autónoma de Puebla, Ciudad Universitaria, 72570 Puebla, PUE Mexico

**Keywords:** BTEX sorption, SBA-15, Mg/Al oxides, Nanoporous composite

## Abstract

Benzene, toluene, ethylbenzene, and *p*-xylene (BTEX) are hazardous volatile organic compounds mostly released from fuel combustion, paint gas emissions, and biomass burning. In this work, it is studied the BTEX sorption influence on the surface reactivity of a new kind of nanoporous composite, prepared via an in situ functionalization of SBA-15 with a Mg–Al calcined hydrotalcite (HT_C_). During its preparation, Mg/Al mixed oxides are indeed formed and dispersed on the SBA-15 surface with non-blockage porosity. Furthermore, the physicochemical surface properties are exalted from its precursors and it is synergistically favorable for the BTEX sorption at low pressure and temperature.

## Introduction

Air pollution has become a notorious worldwide issue of major significance with an arising of uncontrolled pollutant emissions originated by several chemical processes that modify both air quality and natural cycles. The volatile organic compounds (VOC) that can change from liquid to gas at room temperature are highly toxic pollutants. Household and industrial solvents, paints and the burning of fossil fuels are the main sources of VOC emissions. They contribute to photochemical smog in big cities and have an impact on the environment and health of humans [1]. Although there are several studies on VOC catalytic elimination [2, 3], the sorption process is always preferred due to the less cost and recovering of the VOC [4]. For instance, metal–organic frameworks (MOFs), with a large surface area (5870 m^2^/g of BET area) and a 1.85 cm^3^/g total pore volume, have proved to be an effective choice for VOC removal, especially for those molecules presenting steric hindrance as xylenes and ethylbenzene [5]. However, using MOFs as adsorbents is still an unaffordable choice in terms of their preparation costs. Micro and mesoporous materials based on SiO_2_ and functionalized with Ag or Cu has been studied to sorb benzene, toluene, and *p*-xylene (BTX) [6] showing that toluene and *p*-xylene adsorption can be stopped in the case of Cu/SiO_2_, because of a steric hindrance due to an ink-bottle pore shape of such material. BTX sorption it was also carried out by using amine modified KIT-6 and SBA-15 presented highly adsorption increasing with an increase in the pressure [7]. However, a poor benzene adsorption was observed due to the hydrophobicity of the KIT-6 whereas *m*-xylene and toluene presented stronger hydrophobic interaction with the amine-modified material. In the same way, a SBA-15, functionalized with polyvinyl benzene [7] or phenyltriethoxysilane [8], proves to be an excellent VOC hydrophobic adsorbent of toluene, benzene and cyclohexane with no steric hindrance. Cobalt supported on a calcined hydrotalcite was used as a catalyst in the toluene oxidation using the memory effect of Mg–Al hydrotalcite [9]. Hydrotalcites are lamellar materials showing basic properties without significant specific surface area and mesoporosity [10]. Hence, large molecules cannot reach the most of the anchorage sites. If an anionic clay is combined with a mesoporous material (i.e. SBA-15), a great adsorbent can be synthesized [11, 12]. For instance, the SBA-15 is a mesoporous material with a specific surface area larger than 700 m^2^/g and a 0.8 cm^3^/g of pore volume, both are ideal characteristics for a VOC adsorbent. However, the SBA-15 exhibits poor surface reactivity due to a reduced number of anchor sites (silanol groups). In this paper, the SBA-15 surface functionalized with a Mg–Al calcined hydrotalcite (HTc) is carried out. After the calcination of this nanocomposite, Al–Mg-O mixed oxides are dispersed on the SBA-15 surface, and the modification of its textural properties occurs. Indeed, HTc presents a higher chemical reactivity than that of the SBA-15, but a much lower specific area (ca. 74 m^2^/g) and a very small mesoporous volume (ca. 0.20 cm^3^/g). The accurate combination of such materials, with non-organic functionalization, can be readily performed to produce a nanoporous composite with physicochemical properties that have a synergistic influence on the benzene, toluene, ethylbenzene, and xylene sorption.

## Experimental

### Material preparation

#### Sba-15

The SBA-15 preparation was carried out following the methodology proposed by Zhao et al. [13]. In a polyethylene bottle, 16 g of template, Pluronic 123 (EO)_20_(PO)_70_(EO)_20_ (Sigma-Aldrich), were mixed with 474 mL of a 2 M HCl (J. T. Baker, 37%) solution. The mixture remained under stirring at room temperature until the apparent dissolution of the template. Subsequently, 34.4 mL of tetraethylortosilicate (TEOS, Sigma-Aldrich, 98%) were gradually added into this polyethylene bottle and remaining under stirring at room temperature another 24 h. The recipient was treated at 95 °C for 72 h into an oven. The solid was thus recovered by decantation, washed with distilled water and dried at 70 °C. An amount of this sample was calcined at 550 °C in air for 6 h to eliminate the organic template.

#### Mg/Al mixed oxides

The preparation of a Mg/Al hydrotalcite with a 2:1 mol ratio was achieved by using a microwave-assisted coprecipitation method to obtain a material whose formulation is Mg_6_Al_3_(OH)_18_NO_3_·4H_2_O [14]. A 1.5 M solution was prepared from Mg(NO_3_)_2_·6H_2_O and Al(NO_3_)_3_·7H_2_O, (both from Aldrich, 98%). A precipitating solution of NH_4_OH (2 M) was also prepared. Both solutions were dropwise into a flask under stirring and remaining a constant pH 9. The coprecipitated salts were stirred for 24 h at room temperature. The solid was recovered by filtration, washed and dried at 70 °C. Finally, the dried white solid was calcined at 550 °C for 6 h to obtain the (Mg–Al-O) mixed oxides, the sample was labeled as HTc.

#### SBA-15/HT_C25_ composite

The composite was prepared by combining a suitable amount of SBA-15 with a Mg–Al nitrated hydrotalcite to obtain a 80/25 wt% nominal ratio of the calcined composite [11]. 80 wt% of non-calcined SBA-15 was placed into a flask containing 25 mL of distilled water and dispersed by stirring during an hour. Subsequently, the complementary amount of Mg–Al hydrotalcite (ca. 20 wt%) was prepared on the SBA-15 dispersion following the same procedure mentioned in the above section. The dried composite was calcined at 550 °C for 6 h and labeled as SBA-15/HT_C25_.

### Materials characterization

SBA-15, HT_C_, and SBA-15/HT_C25_ samples were characterized by X-ray diffraction (XRD) by using a Bruker D8 equipment with Cu*Kα* radiation, at scanning ranges from 0.7° to 3° (2*θ*) for small angles and from 5° to 70° (2*θ*) for wider angles. Nitrogen adsorption at 77 K was carried out with a Micromeritics ASAP 2010 equipment between relative accuracy ranges, *P/P*_*0*_, from 0.06 up to 0.99 with 0.015 increases. Previously, the samples were degassed at 90 °C for 10 h until a 0.05 mmHg vacuum pressure was reached.

### BTEX adsorption tests

The adsorption of benzene, toluene, ethylbenzene or *p*-xylene (BTEX) was carried out by using a SHIMADZU GC-14A gas chromatograph with a flame ionization detector (FID). A stainless steel chromatographic column (0.6 in diameter and 50 cm long) was packed with 0.1 g of each sample mixed with 0.2 g of ground glass, previously sieving (80/50 mesh). During chromatographic analyses, a high-purity He (99% chromatographic degree) was used as carrying gas (30 cm^3^/min). A pretreatment of samples with He was realized at 300 °C for 1 h. Table 1 shows the temperature intervals of the chromatograph devices during analyses, considering the VOC physical properties, Table 2. On each adsorption test, 1 µL of VOC was injected.

**Table 1:** Temperature intervals for chromatograph devices

	Temperature °C				
	1st	2nd	3rd	4th	5th
Injector	305	270	235	195	160
Furnace	300	260	225	185	150
Detector	315	285	245	205	170

**Table 2:** Physical properties of VOC [15]

VOC	Molecular weight (MW) g/mol	Kinetic diameter (σ) nm	Ionizing potential (P) Ev	Radii (d) nm	Boiling temperature °C
Benzene	78.11	0.65	9.2–9.7	0.73	80.1
Toluene	92.14	0.65/0.89	8.8	0.835	110.6
*p*-Xylene	106.16	0.98/1.05	8.5	0.94	144
Ethylbenzene	106.16	0.98	8.5	0.94	136

#### Equilibrium models of adsorption isotherms

The experimental data were used to model two popular adsorption isotherm equations: Freundlich and Langmuir. The Freundlich isotherm equation [16] was used as follows:

1 $$\log a = \log {k_f} + \frac{1}{n}\log p$$

where *p* is the equilibrium pressure when gas moles are adsorbed at temperature T in Kelvin degrees (K), *a* is the adsorption capacity at equilibrium with the adsorbent, *k*_*f*_ is Freundlich equilibrium constant that shows the adsorption capacity and the adsorbate affinity by the adsorbent, and *1/n* is defined as the rate of adsorbate saturation. Likewise, the Langmuir model isotherm equation [16] was used as follows.

2 $$\frac{P}{a} = \frac{1}{{K{a_m}}} + \frac{P}{a_m}$$

where *P* is the system pressure, *a* is the adsorbed volume at a certain pressure, *a*_*m*_ is the maximum monolayer volume that the surface can adsorb at equilibrium and *K* is the value of Langmuir constant. Both adsorption isotherm equations were fitted using experimental data with correlation coefficients greater than 0.9 for all isotherms. It is also known that at lower pressure, Also, Henry isotherm is a short form of the Langmuir isotherm, represented as follow:

3 $$a = {K_H}P$$

where *K*_*H*_ is the Henry constant.

#### Isosteric heat of adsorption

The isosteric heat of adsorption (*q*_*st*_) was estimated by using adsorption isotherms from the VOC at different temperatures. Isosteric heat of adsorption is the most used property for calculating fixed beds, which is given by the Clausius–Clapeyron equation 3 [6].

4 $${\frac{\partial p}{\partial T}_a} = \frac{{{q_{st}}\left( a \right)}}{{R{T^2}}}$$

The heat of adsorption can be determined from the isotherms acquired at different temperatures.

## Results and discussion

### X-ray diffraction

**Fig. 1:**
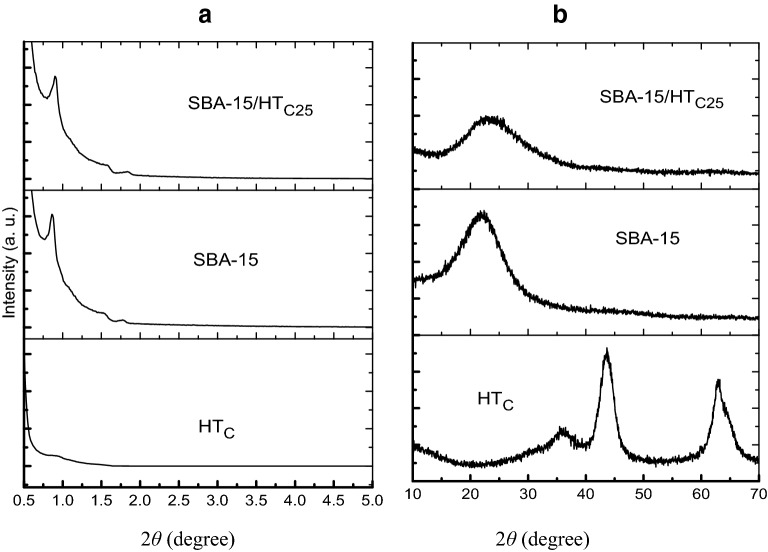
Diffractograms at small (**a**) and wider (**b**) angles

The diffractograms at small angles of the calcined hydrotalcite (HT_C_), SBA-15, and SBA-15/HT_C25_, respectively, were recorded between 0.5° and 5° 2*θ*, Fig. 1a; the (100) diffraction signal it is observed at 0.87° 2*θ* and it is characteristic of an interlayer distance of 10.1 nm, of the SBA-15 nanostructure. A similar, peak diffraction is observed at 0.90° 2*θ*, which belongs to an interlayer distance of 9.8 nm. As expected, the HT_C_ shows no diffraction peaks at small angles. Instead, the recorded diffractogram at wider angles (between 5° y 70° 2*θ*) for the HTc, Fig. 1b, shows the characteristic peaks of the periclase-type structure that belongs to the mixed oxides, Mg–Al-O, at 42° and 65° 2*θ* (JCPDS 00-045-0946). For the SBA-15 and the composite diffractograms, a rather wide shoulder is observed between 15° and 30° 2*θ*. This kind of polycrystalline diffractogram is usually associated with the amorphous silica (JCPDS 00-076-912) that is the main component of the SBA-15 walls. However, no diffraction signal relating to the mixed oxides (Mg–Al-O) are observed for the composite, SBA-15/HT_C25_. Therefore, small Mg–Al-O particles may be homogeneously dispersed on the SBA-15 nanoporous surface [11].

### Nitrogen adsorption

**Fig. 2:**
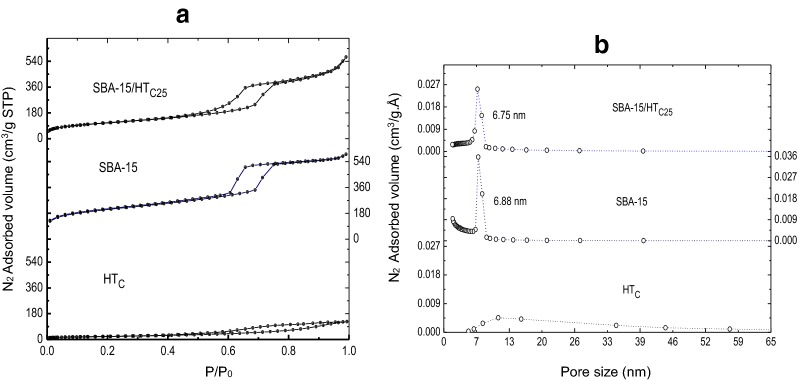
N_2_ adsorption isotherms (**a**) and pore size distribution (**b**) of the HT_C_, SBA-15, and SBA-15/HT_C25_

In Fig. 2a and b, are respectively represented the nitrogen adsorption–desorption isotherms and the pore diameter distribution for the SBA-15, calcined hydrotalcite (HT_C_) and SBA-15/HT_C25_ composite. Each isotherm is type IV, according to the IUPAC classification, indicating that materials present mainly mesoporous. The SBA-15 and SBA-15/HT_C25_ isotherms show an H1 hysteresis loop [17] at P/P_0_ ranging from 0.5 to 0.75, Fig. 2a; it is associated to the mesoporous materials having tubular porous whit a narrow size distribution. Nevertheless, the HT_C_ show an H3 hysteresis loop with a relative pressure interval from 0.64 to 0.90, relating to the porous with laminar structure, as in clays [18].

The SBA-15 and SBA-15/HT_C25_ composite pore diameter distributions are rather similar as shown in Fig. 2b. As N_2_ adsorption–desorption isotherms present similar form curves, they can also be structurally analogous, denoting no mesoporous blocking. The SBA-15 presents a 6.88 nm main pore diameter with a slight decreasing in the composite one (6.75 nm). This small difference (0.11 nm) can be related to the particles of the Mg/Al mixed oxides that are casting the SBA-15 pores. The specific surface areas were estimated by using the BET method (Brunauer, Emmett and Teller) and the pore diameter distributions were computed by using the BJH method (Barrett, Joyner and Halenda) applied to the nitrogen desorption isotherm data [18], Table 3. BET surface area decreased (ca. 32%) in the composite, 402 m^2^/g, in comparison to the SBA-15, 720 m^2^/g; still, it is much bigger than that determined in the HTc (74 m^2^/g). Besides, the wall thickness increased from 4.5 nm (SBA-15) to 4.8 nm (SBA-15/HT_C25_). Therefore, the Mg/Al mixed oxides can form a small layer on the SBA-15 wall surface and the pore size and interlayer parameters are diminished. Moreover, the basic pH used during the composite preparation may contribute to the loss of the textural properties. Furthermore, the microwave radiation used during synthesis might also have caused a decreased specific BET surface area [11]. Still, the narrow distribution of the average pore diameter and the pore volume remain, Fig. 2b, with an increment of the specific surface area and pore volume.

**Table 3:** Textural properties of the materials determined from the results of adsorption analysis of N_2_ at 77 K

Sample	BET specific surface (m^2^/g)	Pore volume (cm^3^/g)	Pore diameter (nm)	*d*_(100)_ (nm)	WT (nm)
HT_C_	74	0.19	Broader (1–65)	–	–
SBA-15	720	0.80	6.88	10.10	4.5
SBA-15/HT_C25_	402	0.83	6.75	9.80	4.8

*WT* wall thickness determined as [(2*d*100/(3)^0.5^) − pore diameter]

### BTEX adsorption isotherms

**Fig. 3:**
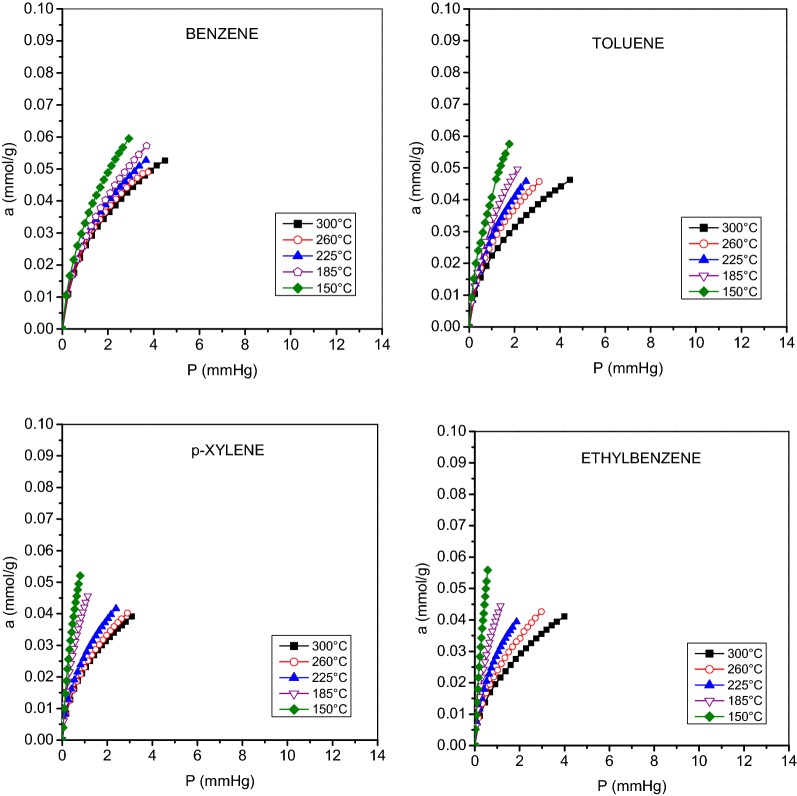
Benzene, toluene, *p*-xylene, and ethylbenzene adsorption isotherms for SBA-15

BTEX adsorption isotherms for the SBA-15 are shown in Fig. 3. In all isotherms, BTEX adsorption is carried out at pressures lower than 5 mmHg because the SBA-15 material has a very wide pore diameter (6.88 nm, shown in Table 4), thus no high pressures are required. The pattern of every isotherm is quite similar to each other. Indeed, the higher the temperature, the lower VOC adsorption occurs. Besides, the adsorbed volume ranges from 0.05 to 0.06 mmol_VOCs_/g_SBA-15_ at 150 °C. The adsorbate–adsorbent interaction depends on the shape of the isotherm. In these cases, the isotherms are concave. Therefore, as the isotherm presents a concave profile, the BTEX adsorption is ensured, thus, it is confirmed that the SBA-15 uptake of the BTEX compounds favorably. Besides, the lower the temperature, the greater BTEX amount is adsorbed. Furthermore, if the molecular weight of benzene derivatives increases, the adsorption pressure diminishes and its ramification plays a slight role in the uptake amount as a temperature function. Indeed, the ethylbenzene shows higher temperature dependence, than that observed on the benzene adsorption.

**Table 4:** Freundlich equation parameters for volatile organic compound (VOC) adsorption in SBA-15, HT_C_, and SBA-15/HT_C25_

VOCs	T (°C)	SBA-15			HT_C_			SBA-15/HT_C25_		
		*k*_*f*_	*1/n*	*R*_*f*_	*k*_*f*_	*1/n*	*R*_*f*_	*k*_*f*_	*1/n*	*R*_*f*_
Benzene	300	0.024	1.884	0.994	0.017	2.325	0.998	0.03	1.658	0.994
	260	0.025	1.858	0.99	0.016	2.187	0.997	0.029	1.708	0.994
	225	0.027	1.848	0.994	0.017	2.291	0.993	0.032	1.721	0.995
	185	0.027	1.738	0.998	0.017	2.281	0.997	0.033	1.633	0.995
	150	0.032	1.667	0.997	0.018	2.205	0.999	0.039	1.555	0.995
Toluene	300	0.021	1.949	0.999	0.016	2.305	0.994	0.024	1.523	0.987
	260	0.025	1.829	0.995	0.014	2.201	0.993	0.028	1.727	0.995
	225	0.027	1.732	0.995	0.013	2.139	0.997	0.031	1.663	0.996
	185	0.031	1.533	0.996	0.014	2.193	0.999	0.035	1.549	0.995
	150	0.041	1.575	0.998	0.019	2.159	0.995	0.046	1.314	0.988
*p*-Xylene	300	0.022	1.893	0.997	0.011	2.095	0.988	0.027	1.732	0.997
	260	0.022	1.819	0.998	0.007	2.315	0.994	0.029	1.682	0.996
	225	0.026	1.786	0.997	0.013	2.206	0.997	0.034	1.541	0.994
	185	0.044	1.472	0.992	0.016	2.155	0.998	0.038	1.442	0.995
	150	0.069	1.19	0.977	0.019	2.16	0.995	0.047	1.353	0.999
Ethylbenzene	300	0.02	1.951	0.999	0.014	2.152	0.976	0.026	1.764	0.999
	260	0.023	1.85	0.999	0.015	2.274	0.981	0.027	1.705	0.999
	225	0.028	1.723	0.995	0.012	2.155	0.997	0.03	1.611	0.998
	185	0.042	1.527	0.99	0.015	2.201	0.998	0.039	1.391	0.989
	150	0.091	1.213	0.997	0.018	2.193	0.994	0.055	1.364	0.989

Where *T* is the experimental temperature in K, *k*_*f*_ (mmHg^−1^) is the value of the Freundlich constant, 1/*n* is the empirical constant, and *R*_*f*_ is the correlation factor

**Fig. 4:**
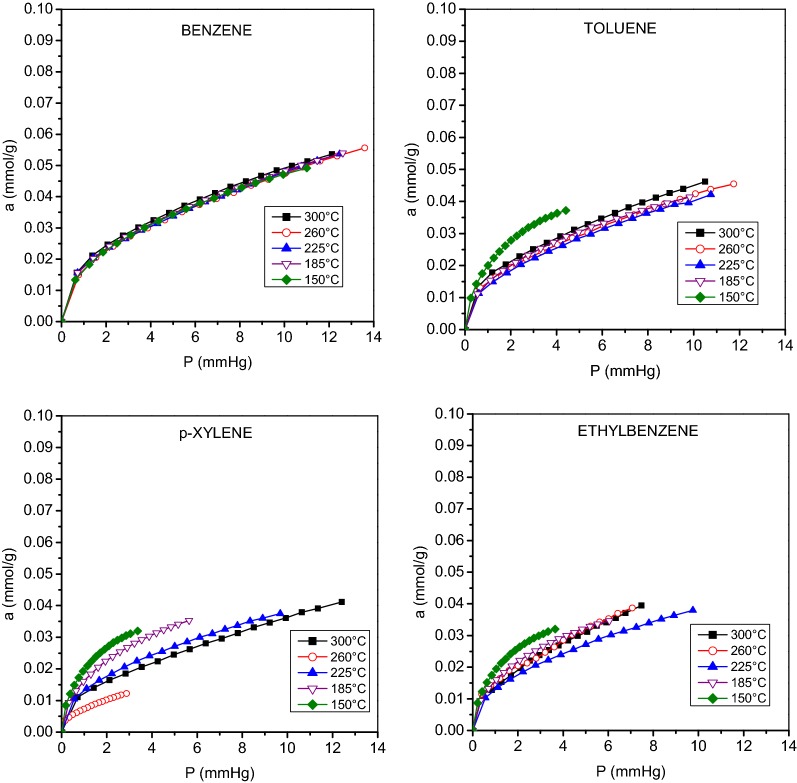
Benzene, toluene, *p*-xylene, and ethylbenzene adsorption isotherms for HT_C_

BTEX adsorption isotherms of Mg/Al hydrotalcite (previously calcined at 550 °C) are shown in Fig. 4. Contrary to that observed on the SBA-15, the BTEX uptake on the HTc occurs at higher pressure. Also, as benzene is a non-polarized hydrophobic molecule and the HTc presents a hydrophilic and low BET specific surface area, no BTEX uptake differences are observed among the temperatures. Still, a major benzene amount is retained on the HT_C_, and the *p*-xylene represents the derivate with the lowest adsorbed amount. This behavior is relating to the steric hindrance due to the poor porosity and specific surface area of HT_C_ although, a favorable adsorbate-adsorbent interaction is observed, as the isotherms are concave. Furthermore, the increased temperature causes no significant difference in the adsorption pattern. However, the BTEX adsorption is more advantageous at 150 °C than at the other temperatures, suggesting the physisorption governs this process. Unlike SBA-15, VOC adsorption is carried out at pressures higher than 10 mmHg in the HT_C_ as it has a low surface temperature and micropores that prevent BTEX molecules sorption.

**Fig. 5:**
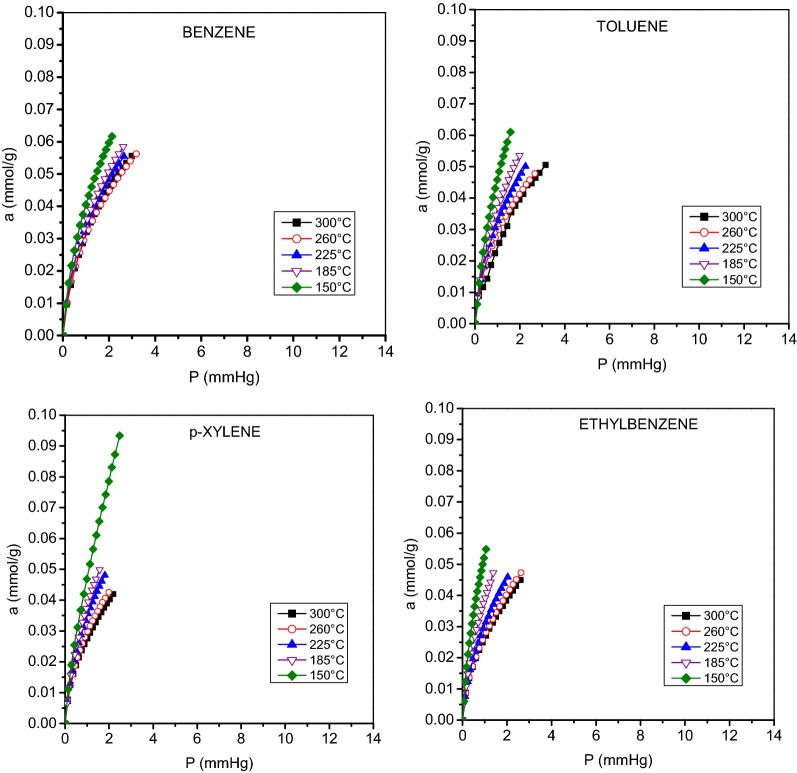
Benzene, toluene, *p*-xylene, and ethylbenzene adsorption isotherms for SBA-15/HT_C25_

In Fig. 5 is showed the BTEX adsorption isotherms for the SBA-15/HT_C25_ composite. These isotherms have a similar behavior to that of the SBA-15, and again, the most favorable temperature for adsorption is 150 °C. However, this composite has a far higher adsorption level than the one observed in the precursor materials. Indeed, HT_C_ and SBA-15 combination boosts VOC adsorption since there is a higher surface interaction owing to the presence of hydrotalcite and a similar mesoporosity to that of SBA-15. Both properties, contribute to a more effective internal diffusion of VOCs. Furthermore, the isotherms show that the composite has a higher affinity to *p*-xylene with an adsorbed volume of 0.095 mmol_VOCs_/g_SBA-15/HTC25_ and the ethylbenzene is the compound that has the least affinity with an adsorbed volume of 0.055 mmol_VOCs_/g_SBA-15/HTC25_. Hence, no steric hindrance is promoted. Moreover, the isotherms are concave and represent a favorable adsorbate-adsorbent interaction and the increasing of temperature diminishes the BTEX amount sorption. This is readily explained due to the volatile properties of the compounds that are used, as very low vapor pressures.

In the SBA-15/HT_C25_, VOC adsorption is carried out at pressures lower than 3 mmHg. This means that the pore diameter (6.75 nm) is large enough to prevent an increased relative pressure. However, the increased amount of adsorbed VOCs is higher than in the SBA-15. Hence, it occurs a synergetic adsorbent interaction, as the Mg/Al mixed oxides can be well dispersed on the SBA-15 surface [11] with no steric hindrance.

#### Freundlich model

**Fig. 6:**
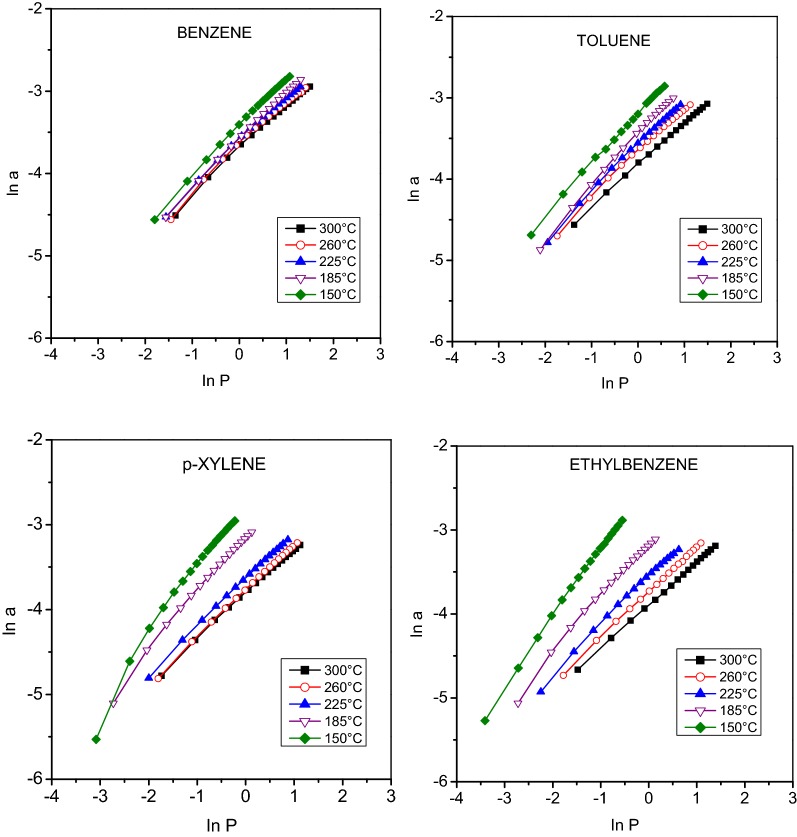
Benzene, toluene, *p*-xylene, and ethylbenzene adsorption isotherms with the Freundlich model for SBA-15

The Freundlich model describes the equilibrium between the solid surface and the BTEX as a multilayer adsorption process. The experimental data obtained from the linear regression of such as isotherms summarized in Table 4 and Fig. 6, respectively. Freundlich equation is an empirical model that is used to estimate the adsorption behavior of BTEX adsorbed on the nanoporous composites. Although the BTEX Freundlich isotherms, by using the SBA-15 as the adsorbent, Fig. 6, are straightforwardly adjusted to this model, the linearity of *p*-xylene isotherm at 150 °C is slightly curved, still, the correlation coefficient, *R*_*f*_ = 0.997, shows a marginal error. The adsorption capacity, *k*_*f*_, increases as the adsorption temperature decrease and if the temperature is lower than 300 °C, *k*_*f*_ also augments with the adsorbate molecular weight: ethylbenzene > p-xylene > toluene > benzene. Thus, the physisorption is the main adsorption process. SBA-15 is an adsorbent, with an excellent specific surface area and narrow porous size that diminishes the steric hindrance of the adsorbates. However, the benzene adsorption is constrained by the SBA-15 surface adsorption reactivity, as it should be energetically controlled by the interaction of the molecules with the SBA-15 pore walls and the number of accessible silanol groups [19].

**Fig. 7:**
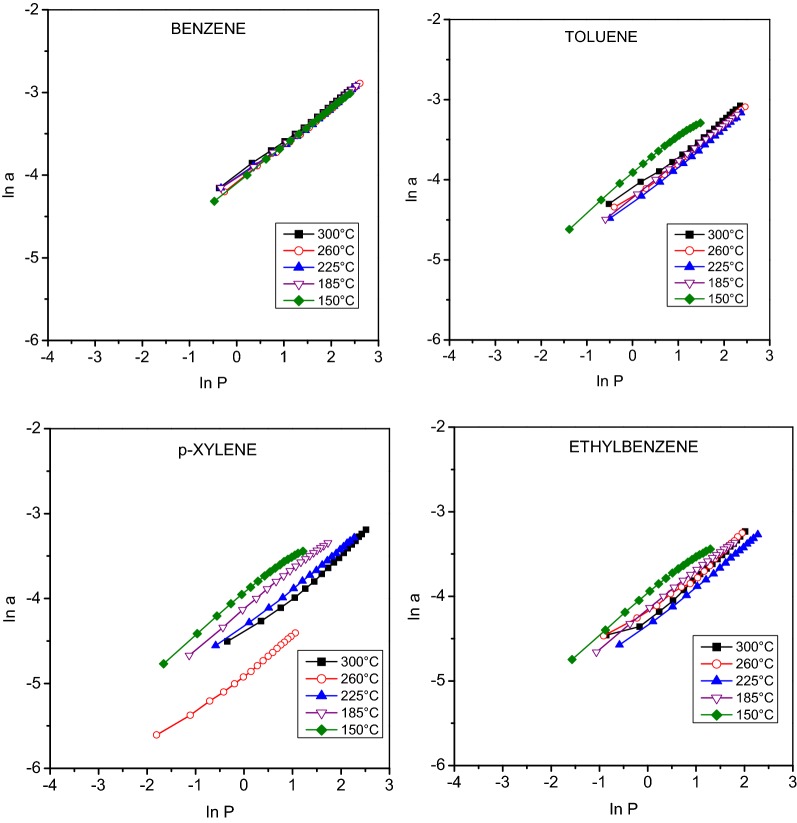
Benzene, toluene, *p*-xylene, and ethylbenzene adsorption isotherms with the Freundlich model for HT_C_

If the HTc is employed as adsorbent the benzene isotherms are perfectly fitted to the Freundlich model at all temperatures, Fig. 7, indicating no influence of the temperature adsorption during the benzene uptake as the *k*_*f*_ values remain almost unchanged, Table 4. Toluene isotherms are mostly completely fitted (in all experimental data) with a slight non-linearity at the boundary temperatures (150 and 300 °C respectively). Furthermore, the Freundlich model, at 300 and 260 °C, for *p*-xylene and ethylbenzene, shows more variation on the fitting data. However, in all cases, the *R*_*f*_ is an indication of an acceptable fitting.

**Fig. 8:**
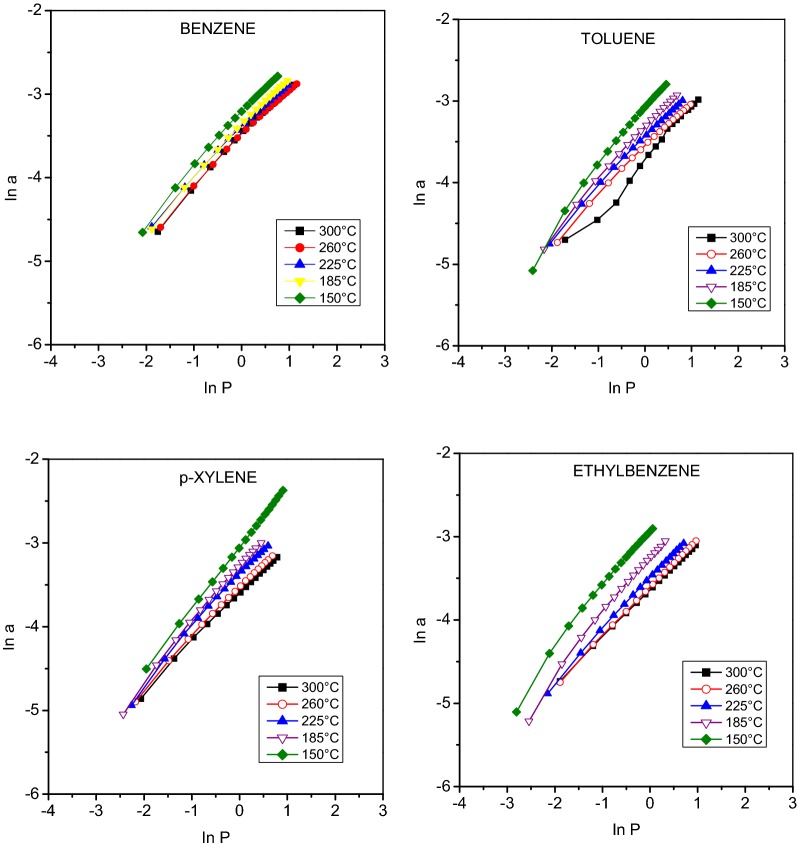
Benzene, toluene, *p*-xylene, and ethylbenzene adsorption isotherms with the Freundlich model for SBA-15/HT_C25_

In Fig. 8 is showed the adsorption isotherms with the Freundlich model for the SBA-15/HT_C25_ composite. The following isotherms are not completely fitted to the Freundlich model: (i) toluene isotherms at 150 and 300 °C, (ii) ethylbenzene isotherms at 150 and 185 °C, and (iii) *p*-xylene isotherm at 150 °C.

All the other isotherms are adjusted to this model, Table 4. Indeed, it presents a *k*_*f*_ and 1/n values greater than those shown for its precursors, SBA-15 and HTc. Indeed, the Mg–Al hydrotalcite prepared in situ of the SBA-15 contributes the increasing of surface reactivity.

In most of the experiments, as the adsorption affinity diminishes (*k*_*f*_) with the raising of the temperature, as the physisorption is the governing adsorption process, which allows the adsorbent regeneration and low temperature of BTEX sorption.

#### Langmuir model

**Fig. 9:**
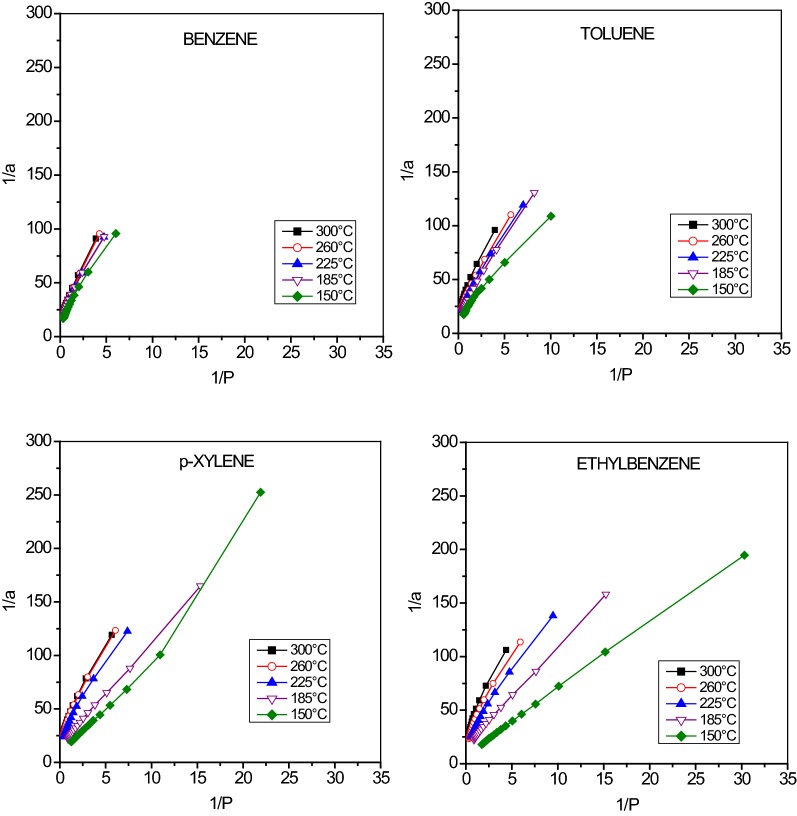
Benzene, toluene, *p*-xylene, and ethylbenzene adsorption isotherms with the Langmuir model for SBA-15

**Fig. 10:**
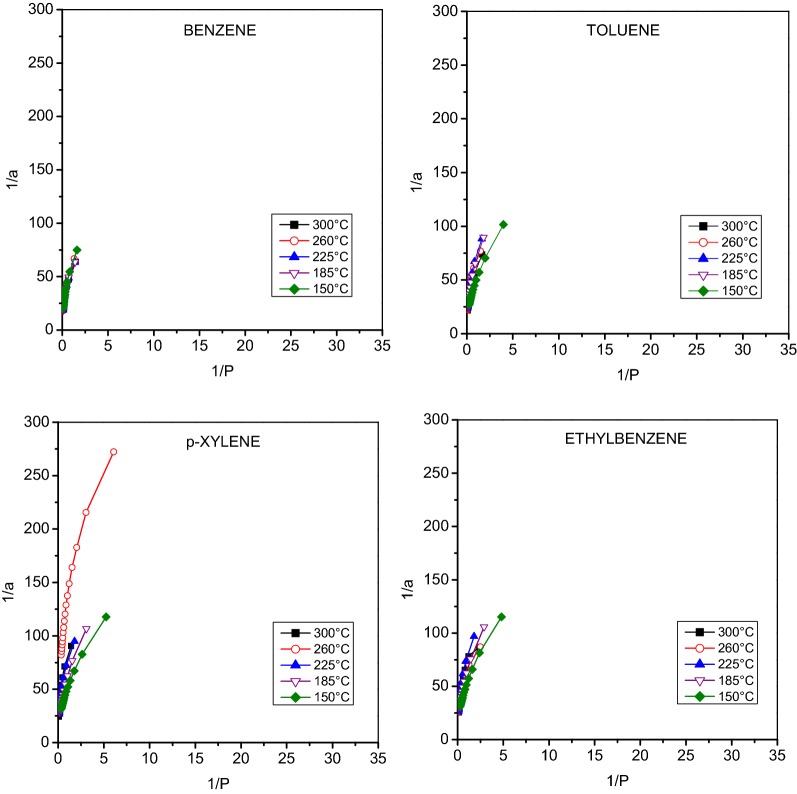
Benzene, toluene, *p*-xylene, and ethylbenzene adsorption isotherms with the Langmuir model for HTc

**Fig. 11:**
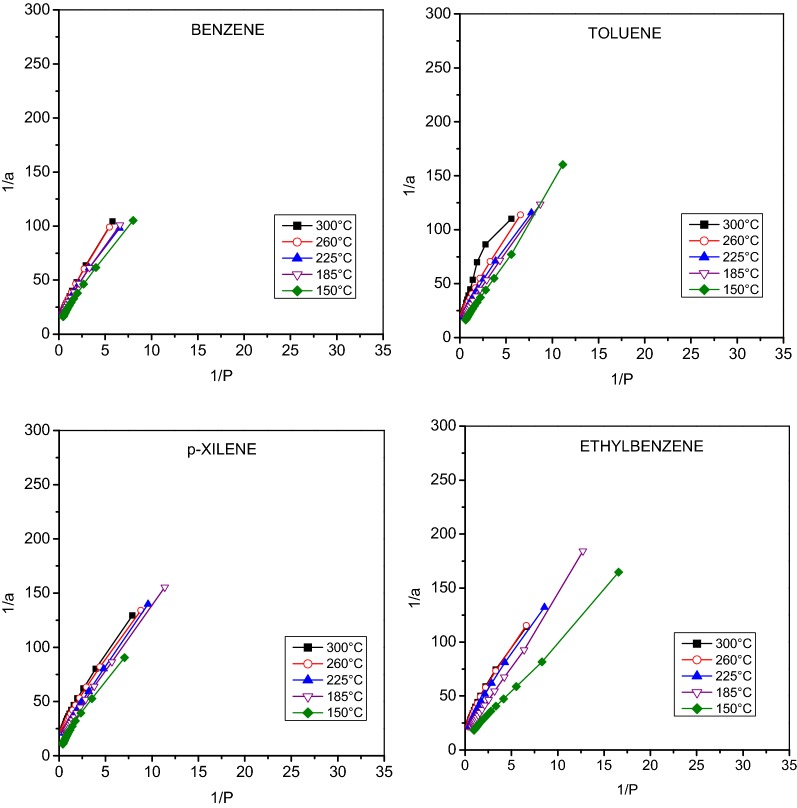
Benzene, toluene, *p*-xylene, and ethylbenzene adsorption isotherms with the Langmuir model for SBA-15/HT_C25_

This model describes the ideal equilibrium between a surface (adsorbent) and a chemical substance in a solution (adsorbate) as a monolayer adsorption. In Figs. 9, 10 and 11 are showed the adsorption isotherms with the Langmuir model for the four BTEX in the SBA-15, HTc and SBA-15/HT_C25_, respectively. In most them, a short curvature is observed, then, these isotherms are neither completely linear, nor completely fitted to the model.

Although the linear correlation coefficient, ***R***_***L***_, tends to have more variations in comparison to the correlation coefficient of the Freundlich model, the experimental data are partly fitted, to the Langmuir model, Table 5, and the physisorption is the predominant adsorption process.

**Table 5:** Langmuir and Henry model parameters for volatile organic compound (VOC) adsorption in SBA-15, HT_C_, and SBA-15/HT_C25_

VOCs	T (°C)	SBA-15				HT_C_				SBA-15/HT_C25_			
		*a*_*m*_	*K*_*L*_	*K*_*H*_	*R*_*L*_	*a*_*m*_	*K*_*L*_	*K*_*H*_	*R*_*L*_	*a*_*m*_	*K*_*L*_	*K*_*H*_	*R*_*L*_
Benzene	300	0.059	0.852	0.05	0.99	0.05	0.586	0.029	0.93	0.068	0.915	0.062	0.993
	260	0.06	0.872	0.052	0.994	0.052	0.468	0.024	0.935	0.067	0.935	0.062	0.993
	225	0.059	1.012	0.06	0.988	0.049	0.577	0.028	0.907	0.067	1.168	0.076	0.991
	185	0.063	0.919	0.058	0.984	0.05	0.559	0.028	0.916	0.071	1.021	0.073	0.992
	150	0.069	1.025	0.071	0.988	0.048	0.564	0.027	0.95	0.081	1.04	0.084	0.996
Toluene	300	0.048	1.026	0.049	0.974	0.042	0.701	0.029	0.906	0.052	1.00	0.052	0.91
	260	0.051	1.179	0.06	0.99	0.041	0.583	0.024	0.898	0.055	1.188	0.065	0.99
	225	0.053	1.271	0.067	0.991	0.039	0.583	0.023	0.922	0.059	1.272	0.075	0.991
	185	0.064	1.075	0.068	0.992	0.04	0.629	0.025	0.95	0.07	1.11	0.077	0.996
	150	0.067	1.538	0.103	0.989	0.039	1.23	0.048	0.974	0.131	0.564	0.074	0.997
p-Xylene	300	0.042	1.335	0.056	0.981	0.036	0.524	0.019	0.885	0.047	1.469	0.07	0.988
	260	0.043	1.316	0.057	0.981	0.011	2.58	0.028	0.898	0.049	1.498	0.074	0.99
	225	0.046	1.514	0.069	0.984	0.035	0.675	0.023	0.922	0.064	1.178	0.075	0.997
	185	0.067	1.509	0.102	0.999	0.036	1.002	0.036	0.962	0.070	1.122	0.079	0.998
	150	0.078	1.117	0.092	0.981	0.033	1.635	0.055	0.972	0.124	0.666	0.082	0.991
Ethylbenzene	300	0.041	1.185	0.048	0.965	0.033	0.987	0.033	0.818	0.048	1.387	0.066	0.977
	260	0.044	1.379	0.06	0.976	0.034	1.089	0.037	0.866	0.051	1.258	0.065	0.981
	225	0.046	1.7	0.078	0.99	0.035	0.644	0.023	0.925	0.054	1.33	0.072	0.989
	185	0.062	1.701	0.105	0.999	0.035	0.974	0.034	0.962	0.081	0.921	0.074	0.998
	150	0.120	1.317	0.16	0.999	0.035	1.535	0.052	0.972	0.101	1.069	0.108	0.998

Langmuir monolayer capacity a_m_ (mmol/g), adsorbent’s maximum adsorption capacity, Henry constants K_H_ (mmHg^−1^), Langmuir K_L_ (mmHg^−1^), and R_L_ linear correlation coefficient

The maximum BTEX amount that the materials can adsorb at the equilibrium is indicated by the monolayer capacity, ***a***_***m***_. Such values confirm that the materials have a higher affinity to benzene and toluene, if the temperature is lower than 300 °C, while *p*-xylene and ethylbenzene have less adsorption affinity if temperature increases. Indeed, with the increasing of the weight molecular and the temperature different pressure and adsorption capacity, *a*, are observed. As expected, at low temperature the BTEX amount adsorption increases and the composite, SBA-15/HT_C25_ exhibits a greater adsorption capacity.

### Isosteric heats of adsorption

**Fig. 12:**
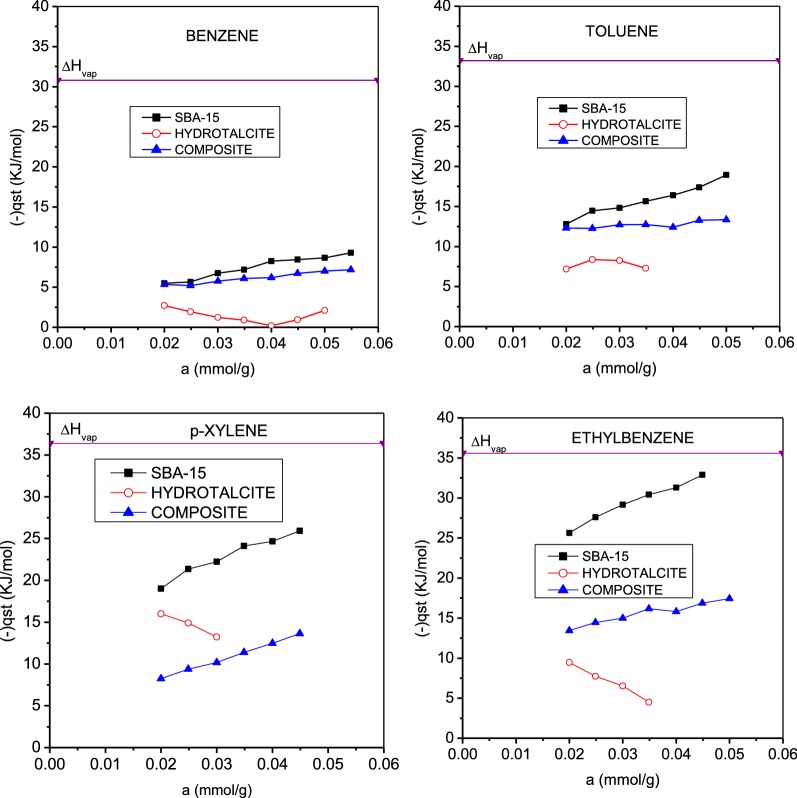
Isosteric heats of adsorption of benzene, toluene, *p*-xylene, and ethylbenzene for SBA-15, HT_C_, and SBA-15/HT_C25_, respectively

As adsorption is an exothermic process, increasing the temperature and keeping the constant pressure enhancement adsorbate desorption. In some systems, physisorption is the predominant process at low temperatures, whereas chemisorption is present at high temperatures [6]. In Fig. 12 are plotted benzene, toluene, *p*-xylene, and ethylbenzene isosteric heats of adsorption in the three materials studied: SBA-15, HTc and SBA-15/HT_C25_. In the four cases, the isosteric heats of adsorption are smaller than the vaporization heat (∆H_vap_) of each one of the VOC adsorbed. This confirms that only a physisorption process is carried out. The values for the SBA-15 and the composite profile have an upward behavior, which shows that they present a homogeneous superficial surface. However, HT_C_ isotherms have a downward behavior, which indicates a heterogeneous superficial surface.

Table 6 displays both the values of standard adsorption energy − ∆U_0_ and the isosteric heat or differential enthalpy, which allow describing the energy changes and determining the spontaneity of the adsorption process on the surface of the material.

**Table 6:** Values of the free standard adsorption energy and the isosteric heats of adsorption

	Benzene		Toluene		*p*-Xylene		Ethylbenzene	
	− ∆U_0_	− q_st_	− ∆U_0_	− q_st_	− ∆U_0_	− q_st_	− ∆U_0_	− q_st_
SBA-15	4.766	9.294	8.997	18.926	6.848	25.921	15.743	32.897
HT_C_	− 1.166	− 2.711	7.538	8.374	13.627	16.013	5.905	9.449
SBA-15/HT_C25_	4.348	7.182	10.926	13.341	2.094	13.631	2.109	17.438

Standard adsorption energy (∆U_0_, kJ/mol) and isosteric heats of adsorption (q_st_, kJ/mol) of volatile organic compounds

The isosteric heat is higher than the free adsorption energy of the VOC thus, the molecules interact strongly with both the neighboring surface and the adsorbate molecules. Still, benzene shows the lowest isosteric heat and may favor a repulsive interaction between the adsorbed benzene molecules [20]. Hence, the adsorption uptake on materials is also lower than that observed for the other adsorbates.

In composite, SBA-15/HT_C25_, the isosteric heat presents values between those measured for the pristine materials, indicating that the surface reactivity has characteristics of both, SBA-15 and HT_C_, respectively. Certainly, the Mg and Al mixed oxides favorably modify the reactivity of the SBA-15 surface and the perseveration of its nanostructure contribute to the BTEX sorption at low temperature, as no steric hindrance occurs.

## Conclusions

The SBA-15/HT_C25_ composite presented a good effective BTEX adsorption performance as the nanostructure and Mg–Al oxides enhance its surface reactivity, improving the BTEX sorption. Therefore, the physicochemical properties of both materials present a synergistic collaboration, in terms of their surface reactivity, that improves the adsorption process. The adsorption isotherms obtained from the adsorbent materials are mainly fitted to the experimental Freundlich adsorption pattern and the VOC are favorably adsorbed, as physisorption process is predominant. Furthermore, these materials can be reused for more time without losing their structural properties and without being affected by adsorption. Composites prepared from SBA-15 and HTc open new ways to produce materials with physicochemical improved properties that enhance the VOC adsorption process without a steric hindrance of the adsorbates.

## References

[1] Agency for Toxic Substances and Disease Registry, ATSDR. (2007), https://www.atsdr.cdc.gov/toxprofiles/index.asp#B. Retrieved 31 August 2017

[2] Kamal MS, Razzak SA, Hossain MH (2016). Catalytic oxidation of volatile organic compounds (VOCs). Atmos. Environ..

[3] He C, Li P, Cheng J, Hao ZP, Xu ZP (2010). A comprehensive study of deep catalytic oxidation of benzene, toluene, ethylacetate, and their mixtures over Pd/ZSM-5 catalyst: mutual effects and kinetics. Water Air Soil Pollut..

[4] Khan FI, Ghoshal AK (2000). Removal of volatile organic compounds from polluted air. J. Loss Prev. Process Ind..

[5] Yang K, Sun Q, Xue F, Lin D Adsorption (2011). Adsorption of volatile organic compounds by metal–organic frameworks MIL-101: influence of molecular size and shape. J. Hazard. Mater..

[6] Hernández MA, Asomoza M, Rojas F, Solís S, Portillo R, Salgado MA, Felipe C, Portillo Y, Hernández F (2010). Trapping of BTX compounds by SiO_2_, Ag–SiO_2_, Cu–SiO_2_, and Fe–SiO_2_ porous substrates. Chemosphere.

[7] Lu H, Cao J, Zhou Y, Zhan D, Chen Y (2013). Novel hydrophobic PDVB/R-SiO_2_ for adsorption of volatile organic compounds from highly humid gas stream. J. Hazard. Mater..

[8] Dou B, Hu Q, Li J, Qiao S, Hao Z (2011). Adsorption performance of VOCs in ordered mesoporous silicas with different pore structures and surface chemistry. J. Hazard. Mater..

[9] Gennequin C, Barakat T, Tidahy HL, Cousin R, Lamonier J-F, Aboukaïs A, Siffert S (2010). Use and observation of the hydrotalcite “memory effect” for VOC oxidation. Catal. Today.

[10] Cavani F, Trifirò F, Vaccari A (1991). Hydrotalcite-type anionic clays: preparation, properties and applications. Catal. Today.

[11] Pérez-Verdejo A, Sampieri A, Pfeiffer H, Ruiz-Reyes M, Santamaría JD, Fetter G (2014). Nanoporous composites prepared by a combination of SBA-15 with Mg–Al mixed oxides. Water vapor sorption properties. Beilstein J. Nanotechnol..

[12] Anunziata O, Martínez M, Beltramone A (2009). Hydroxyapatite/MCM-41 and SBA-15 nano-composites: preparation, characterization and applications. Materials.

[13] Zhao DY, Huo QS, Feng JL, Chmelka BF, Stucky GD (1998). Nonionic triblock and star diblock copolymer and oligomeric surfactant syntheses of highly ordered, hydrothermally stable, mesoporous silica structures. J. Am. Chem. Soc..

[14] Sampieri A, Fetter G, Pfeiffer H, Bosch P (2007). Carbonate phobic (Zn, Mn)-Al hydrotalcite-like compounds. Solid State Sci..

[15] Riddick J, Bunger W, Sakano T (1986). Organic Solvents Physical Properties and Methods of Purification.

[16] Ruthven DM (1984). Principles of Adsorption and Adsorption Processes.

[17] Rouquerol G, Rouquerol J, Sing K (1999). Adsorption by Powders and Porous Solids.

[18] Sing KSW (1998). Adsorption methods for the characterization of porous materials. Adv. Coll. Interface Sci..

[19] Ide M, El-Roz M, De Canck E, Vicente A, Planckaert T, Bogaerts T, Van Driessche I, Lynen F, Van Speybroeck V, Thybault-Starzykb F, Van Der Voort P (2013). Quantification of silanol sites for the most common mesoporous ordered silicas and organosilicas: total versus accessible silanols. Phys. Chem. Chem. Phys..

[20] Zaitan H, Korrir A, Chafik T, Banchi D (2013). Evaluation of volatile organic compound (di-methyl-benzene) removal using adsorption on natural minerals compared to comerical oxides. J. Hazard. Mater..

